# Soil resources and element stocks in drylands to face global issues

**DOI:** 10.1038/s41598-018-32229-0

**Published:** 2018-09-13

**Authors:** César Plaza, Claudio Zaccone, Kasia Sawicka, Ana M. Méndez, Ana Tarquis, Gabriel Gascó, Gerard B. M. Heuvelink, Edward A. G. Schuur, Fernando T. Maestre

**Affiliations:** 1 Instituto de Ciencias Agrarias, Consejo Superior de Investigaciones Científicas, Serrano 115 bis, 28006 Madrid, Spain; 2 Departamento de Biología y Geología, Física y Química Inorgánica, Escuela Superior de Ciencias Experimentales y Tecnología, Universidad Rey Juan Carlos, 28933 Móstoles, Spain; 3 Center for Ecosystem Science and Society, Northern Arizona University, Flagstaff, Arizona 86011 USA; 4 Department of the Sciences of Agriculture, Food and Environment, University of Foggia, via Napoli 25, 71122 Foggia, Italy; 5 Environment Centre Wales, Centre for Ecology & Hydrology, Deiniol Road, Bangor, LL57 2UW UK; 6 Departamento de Ingeniería de Materiales, ETSI Minas, Universidad Politécnica de Madrid, Ríos Rosas 21, 28003 Madrid, Spain; 7 Departamento de Matemáticas, ETSI Agrónomos, Universidad Politécnica de Madrid, Ciudad Universitaria, 28004 Madrid, Spain; 8 Departamento de Edafología, ETSI Agrónomos, Universidad Politécnica de Madrid, Ciudad Universitaria, 28004 Madrid, Spain; 9 Soil Geography and Landscape Group, Wageningen University, 6700 AA Wageningen, The Netherlands; 10 ISRIC - World Soil Information, 6700 AJ Wageningen, The Netherlands

## Abstract

Drylands (hyperarid, arid, semiarid, and dry subhumid ecosystems) cover almost half of Earth’s land surface and are highly vulnerable to environmental pressures. Here we provide an inventory of soil properties including carbon (C), nitrogen (N), and phosphorus (P) stocks within the current boundaries of drylands, aimed at serving as a benchmark in the face of future challenges including increased population, food security, desertification, and climate change. Aridity limits plant production and results in poorly developed soils, with coarse texture, low C:N and C:P, scarce organic matter, and high vulnerability to erosion. Dryland soils store 646 Pg of organic C to 2 m, the equivalent of 32% of the global soil organic C pool. The magnitude of the historic loss of C from dryland soils due to human land use and cover change and their typically low C:N and C:P suggest high potential to build up soil organic matter, but coarse soil textures may limit protection and stabilization processes. Restoring, preserving, and increasing soil organic matter in drylands may help slow down rising levels of atmospheric carbon dioxide by sequestering C, and is strongly needed to enhance food security and reduce the risk of land degradation and desertification.

## Introduction

**Figure 1:**
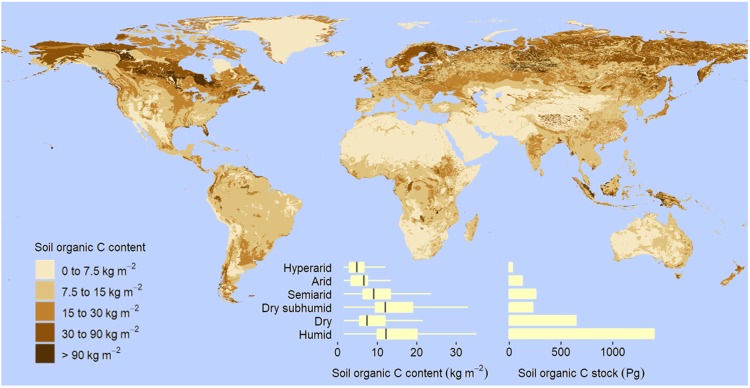
Global distribution of soil organic C content and stock to 2-m depth. Based on the aridity index (AI), or ratio of total annual precipitation to potential evapotranspiration, drylands are divided into hyperarid (AI less than 0.03 mm mm^−1^), arid (AI within the rage from 0.03 to 0.2 mm mm^−1^), semiarid (AI from 0.2 to 0.5 mm mm^−1^), and dry subhumid regions (AI from 0.5 to 0.65 mm mm^−1^). Box, first and third quartile; central horizontal line, median; whisker, 1.5 times the interquartile range, or maximum or minimum if less.

Drylands are regions of the Earth characterized by a water deficit in average climatic conditions, having a ratio of precipitation to potential evaporation, or aridity index (AI), less than 0.65^1–3^. This scarcity of water, caused by low and highly variable precipitation, relatively high temperature, and intense solar radiation, results in a low primary production and very high vulnerability to environmental change^1,2^. According to the most recent and detailed estimate available at global level^4^, drylands cover 66.7 Mkm^2^, or 45% of the Earth’s land surface. Using the dryland subtypes defined in the CGIAR-CSI global aridity database^5^, hyperarid (AI less than 0.03 mm mm^−1^), arid (AI from 0.03 to 0.2 mm mm^−1^), semiarid (AI from 0.2 to 0.5 mm mm^−1^), and dry subhumid (AI from 0.5 to 0.65 mm mm^−1^) regions account for approximately 5.8, 14.0, 16.1, and 8.9% of the Earth’s land area, respectively (Supplementary Table S1 and Fig. S1). Of the global dryland area, approximately 11%, or 7.6 Mkm^2^, is used as cropland and 30%, or 20.0 Mkm^2^, as pasture (Fig. 1, Table 1). In other words, 74% of the global pastures and 50% of the croplands are in drylands, especially in semiarid regions (Supplementary Table S1 and Fig. S1). According to our estimations, drylands are currently inhabited by more than 2.8 billion people, or 39% of the global population (Supplementary Table S1 and Fig. S1). More than 70% of the dryland area is located in developing countries, so drylands are of paramount importance for achieving the global sustainability of current and future human population^1,6^.

**Table 1:** Soil organic C, inorganic C, and total N stocks (mean ± standard deviation) in hyperarid, arid, semiarid, dry subhumid, dry and humid areas.

Land	Organic C (Pg)			Inorganic C (Pg)			Total N (Pg)		
	0–0.3 m	0–1 m	0–2 m	0–0.3 m	0–1 m	0–2 m	0–0.3 m	0–1 m	0–2 m
Hyperarid	11 ± 1	22 ± 1	31 ± 1	20 ± 2	65 ± 3	127 ± 5	1.3 ± 0.1	2.9 ± 0.1	4.5 ± 0.2
Arid	45 ± 3	91 ± 3	127 ± 3	63 ± 2	241 ± 5	487 ± 9	4.9 ± 0.2	10.9 ± 0.2	17.3 ± 0.3
Semiarid	100 ± 2	190 ± 3	259 ± 3	48 ± 2	207 ± 4	456 ± 7	9.3 ± 0.1	19.6 ± 0.2	30.0 ± 0.2
Dry subhumid	91 ± 3	167 ± 4	228 ± 6	15 ± 1	66 ± 2	168 ± 4	7.1 ± 0.2	14.3 ± 0.2	21.4 ± 0.3
Dry	248 ± 6	470 ± 7	646 ± 9	145 ± 4	578 ± 8	1237 ± 15	22.6 ± 0.4	47.7 ± 0.5	73.2 ± 0.6
Humid	502 ± 12	955 ± 19	1401 ± 36	28 ± 3	107 ± 5	321 ± 9	36.1 ± 0.7	72.6 ± 1.1	111.1 ± 1.6
Global	750 ± 15	1425 ± 21	2047 ± 39	173 ± 5	686 ± 10	1558 ± 19	58.6 ± 0.8	120.4 ± 1.3	184.2 ± 1.9

Aridity is forecasted to increase globally due to ongoing global warming and changes in rainfall patterns^7,8^. As a consequence, drylands will degrade and expand to cover 56% of the Earth’s land surface by 2100 under the current path of global warming^7,8^. Dryland expansion and degradation—broadly defined as the loss of functions and services provided by ecosystems over time^9^—threaten the livelihoods of hundreds of millions of people, especially in the developing world^6^, and have long been recognized as a major global environmental, social, and economic issue (e.g., Convention to Combat Desertification adopted by the United Nations in 1994).

Soils, and particularly soil organic matter, are key components of drylands, as of any terrestrial ecosystem, and provide a range of functions and services that have a broad impact on major global issues, such as food security and climate change^10–12^. Globally, soils are the substrate and support for natural vegetation and most of the agricultural production in the world, the natural medium through which water is filtered and held, and one of the largest active carbon (C) reservoirs on Earth^13,14^. Soil organic matter, the fraction of the soil formed by microbial, plant, and animal C compounds at different stages of decomposition, contributes to virtually all soil functions and services by providing a number of benefits, including water-holding capacity and erosion protection^10,15^, which are of particular relevance to drylands.

The most commonly used compendia of dryland soil information, including soil C stock, available at this time date back several decades^1,16–18^. This information lacks uncertainty estimates and is fundamentally based on the delineation of dryland boundaries reported in the second edition of the World Atlas of Desertification, which relied on climatic data collected from 1951 to 1980^3^. Based on more recent and detailed climatic data collected between 1950 and 2000^5^, drylands have expanded under global warming, particularly over the last decades^19,20^, and the current estimate of their extent is about 4% greater than previous estimates^4^. Further, although the biogeochemical cycle of C is closely linked to those of nitrogen (N) and phosphorus (P) in drylands^21^, there are no published global estimates of soil N and P stocks for the world’s drylands.

Here we use the most recent and detailed climatic and soil global geo-databases to provide an updated description of drylands soils and major element stocks, with a focus on C, aimed at serving as a benchmark in the face of future challenges, including population and food security, land degradation, especially erosion and desertification, biodiversity loss, and climate change. For soil properties and C and N stocks, we use WISE30sec, an updated harmonized dataset recently developed^22^. WISE30sec provides soil class and a number of physical and chemical soil properties, including C and N concentrations, for the world at an unprecedented detail, up to a depth of 2 m and with standard deviations, which allows us to provide estimates of C and N stocks in dryland soils together with uncertainties for the first time. For P stocks, we use also the most recently released and detailed maps of the global distribution of different forms of soil P^23^.

## Results

Regosols (i.e., soils with very limited development), Leptosols (i.e., very shallow soils), Arenosols (i.e., sandy soils), and Calcisols (i.e., soils with secondary calcium carbonates) are, in this order, the most common soil groups in drylands, making up about half of their area (Supplementary Table S2). In contrast, the most abundant soils in humid regions are Cambisols (i.e., soils with some development), Acrisols (i.e., acid soils with subsurface accumulation of clays), Leptosols, Ferrasols (i.e., strongly weathered soils with accumulation of oxides), and Podzols (i.e., acid soils with subsurface accumulation of metal-organic compounds) (Supplementary Table S2). Regosols constitute the most common soil group in hyperarid and arid lands, whereas Leptosols and Cambisols are the most abundant soils in semiarid and dry subhumid regions (Supplementary Table S2).

Compared to soils in humid regions, drylands soils generally exhibit better drainage, lower available water storage capacity, coarser texture with larger sand and smaller clay concentrations, and higher bulk density (Supplementary Table S3). Furthermore, dryland soils show markedly higher pH, lower cation exchange capacity, much smaller aluminum saturation, much larger base saturation, higher sodicity, much higher calcium carbonate and gypsum concentrations, and higher salinity (Supplementary Table S3). In general, differences in soil physical and chemical properties between drylands and humid regions tend to be more pronounced with increasing aridity (Supplementary Tables S3).

**Figure 2:**
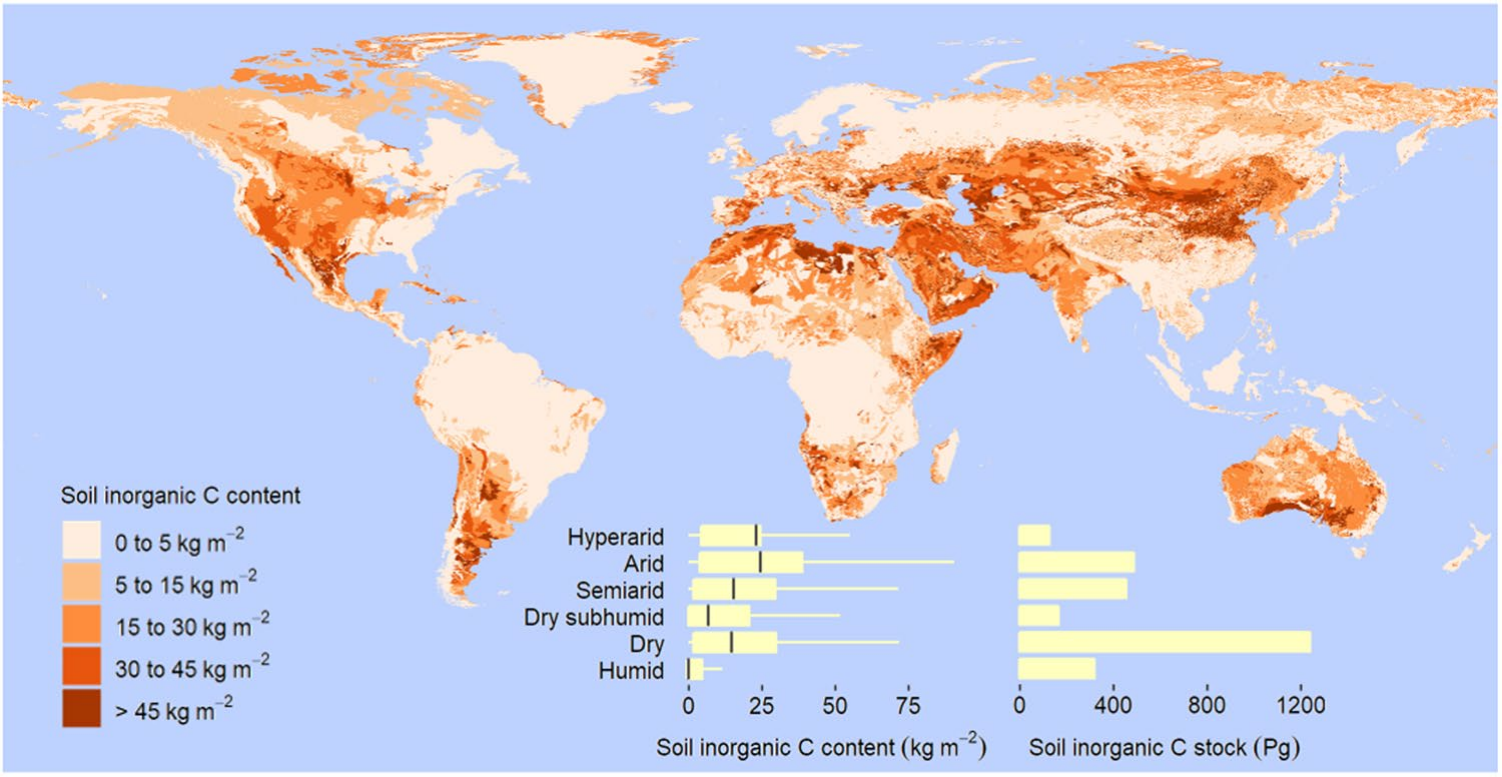
Global distribution of soil inorganic C content and stock to 2-m depth. Based on the aridity index (AI), or ratio of total annual precipitation to potential evapotranspiration, drylands are divided into hyperarid (AI less than 0.03 mm mm^−1^), arid (AI within the rage from 0.03 to 0.2 mm mm^−1^), semiarid (AI from 0.2 to 0.5 mm mm^−1^), and dry subhumid regions (AI from 0.5 to 0.65 mm mm^−1^). Box, first and third quartile; central horizontal line, median; whisker, 1.5 times the interquartile range, or maximum or minimum if less.

Dryland soils have much smaller organic C concentrations than soils in humid regions (Supplementary Table S3). Soil organic C content to any depth up to 2 m increases as aridity decreases from hyperarid to dry subhumid conditions (Table 1, Fig. 1). In particular, mean values of soil organic C content to 2 m vary from 5 kg m^−2^ in hyperarid to 18 kg m^−2^ in dry subhumid regions (Fig. 1). We found that the global amount of soil organic C (mean ± standard deviation) stored in drylands is 248 ± 6 to 30 cm, 470 ± 7 Pg to 1 m, and 646 ± 9 Pg to 2 m, the equivalent of about 32% of the global soil organic C pool (Table 1, Fig. 1). In addition to organic C, dryland soils store vast amounts of inorganic C. In particular, soil inorganic C content to any depth up to 2 m is positively related to aridity, and the amount of soil inorganic C in dryland soils is estimated at 145 ± 4 Pg to only 30 cm, 578 ± 8 Pg to 1 m, and 1237 ± 15 Pg to 2 m; this is about 80% of the global soil inorganic C pool (Table 1, Fig. 2).

**Figure 3:**
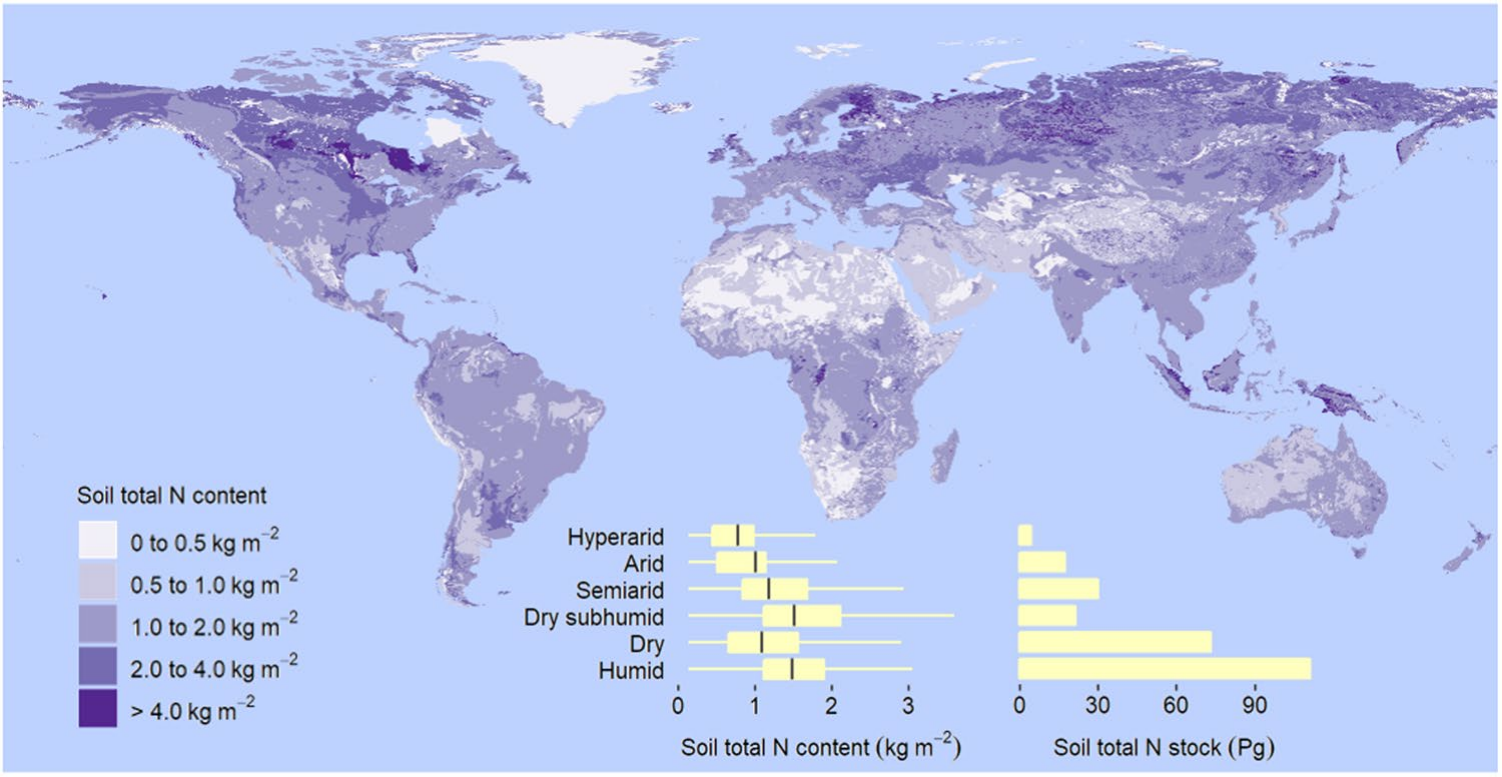
Global distribution of soil total N content and stock to 2-m depth. Based on the aridity index (AI), or ratio of total annual precipitation to potential evapotranspiration, drylands are divided into hyperarid (AI less than 0.03 mm mm^−1^), arid (AI within the rage from 0.03 to 0.2 mm mm^−1^), semiarid (AI from 0.2 to 0.5 mm mm^−1^), and dry subhumid regions (AI from 0.5 to 0.65 mm mm^−1^). Box, first and third quartile; central horizontal line, median; whisker, 1.5 times the interquartile range, or maximum or minimum if less.

**Figure 4:**
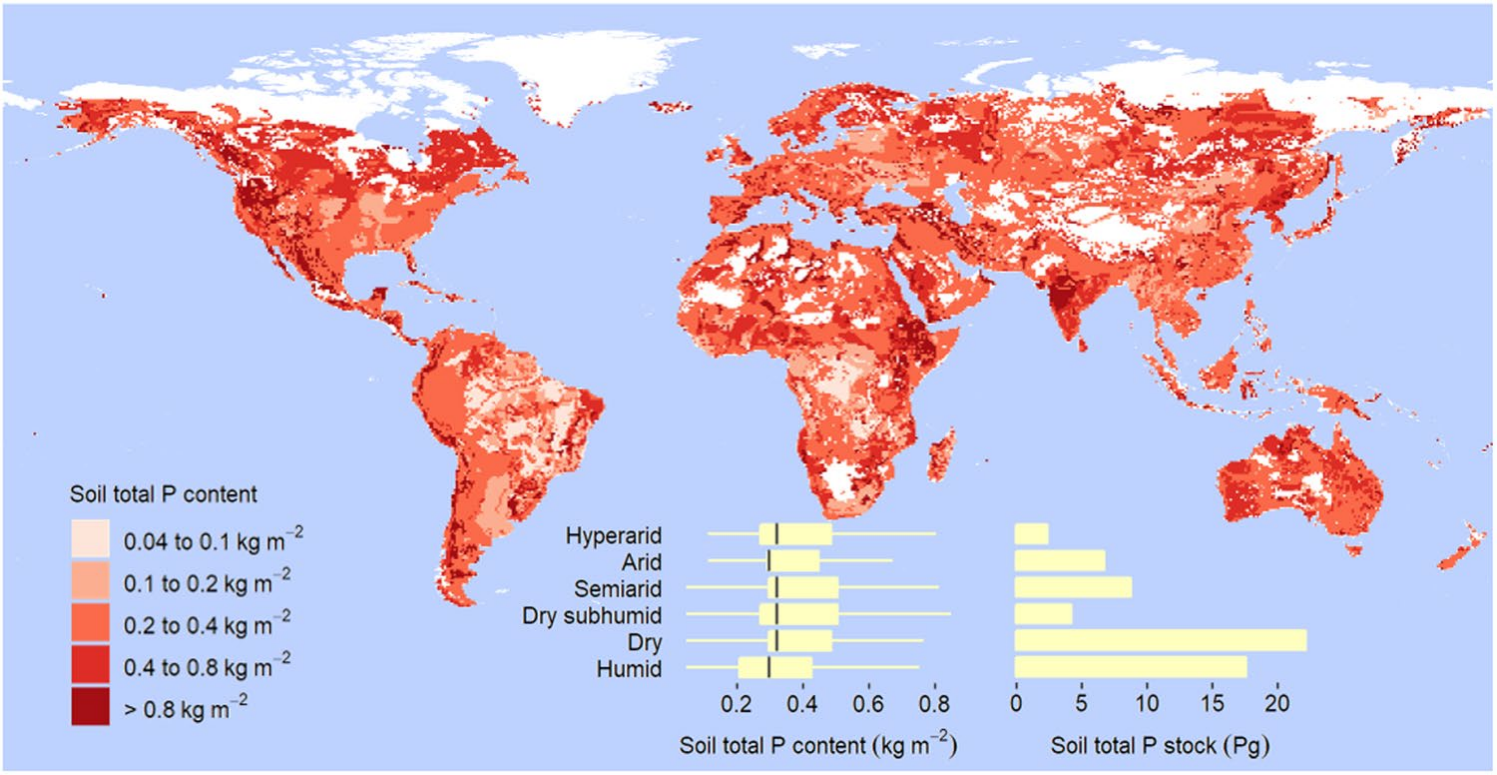
Global distribution of soil total P content and stock to 0.5-m depth. Based on the aridity index (AI), or ratio of total annual precipitation to potential evapotranspiration, drylands are divided into hyperarid (AI less than 0.03 mm mm^−1^), arid (AI within the rage from 0.03 to 0.2 mm mm^−1^), semiarid (AI from 0.2 to 0.5 mm mm^−1^), and dry subhumid regions (AI from 0.5 to 0.65 mm mm^−1^). Box, first and third quartile; central horizontal line, median; whisker, 1.5 times the interquartile range, or maximum or minimum if less.

Similar to what is observed with organic C, the concentration of total N is much smaller in dryland soils than in soils of humid regions (Supplementary Table S3). Also similar to organic C content, total N content in soil increases with decreasing aridity but to a less degree (Table 1, Fig. 3). Dryland soils store 73.2 ± 0.6 Pg of total N to 2 m, about 40% of the global soil N (Table 1, Fig. 3). Unlike organic C and total N, total P content is higher in dryland soils than in soils of humid regions (Fig. 4, Supplementary Table S4). Dryland soils also exhibit larger labile inorganic and apatite P contents, but are poorer in organic, occluded, and secondary mineral P (Supplementary Table S4). Globally, the total P stored in dryland soils to 0.5 m is 22.2 Pg (Fig. 4, Supplementary Table S4).

**Figure 5:**
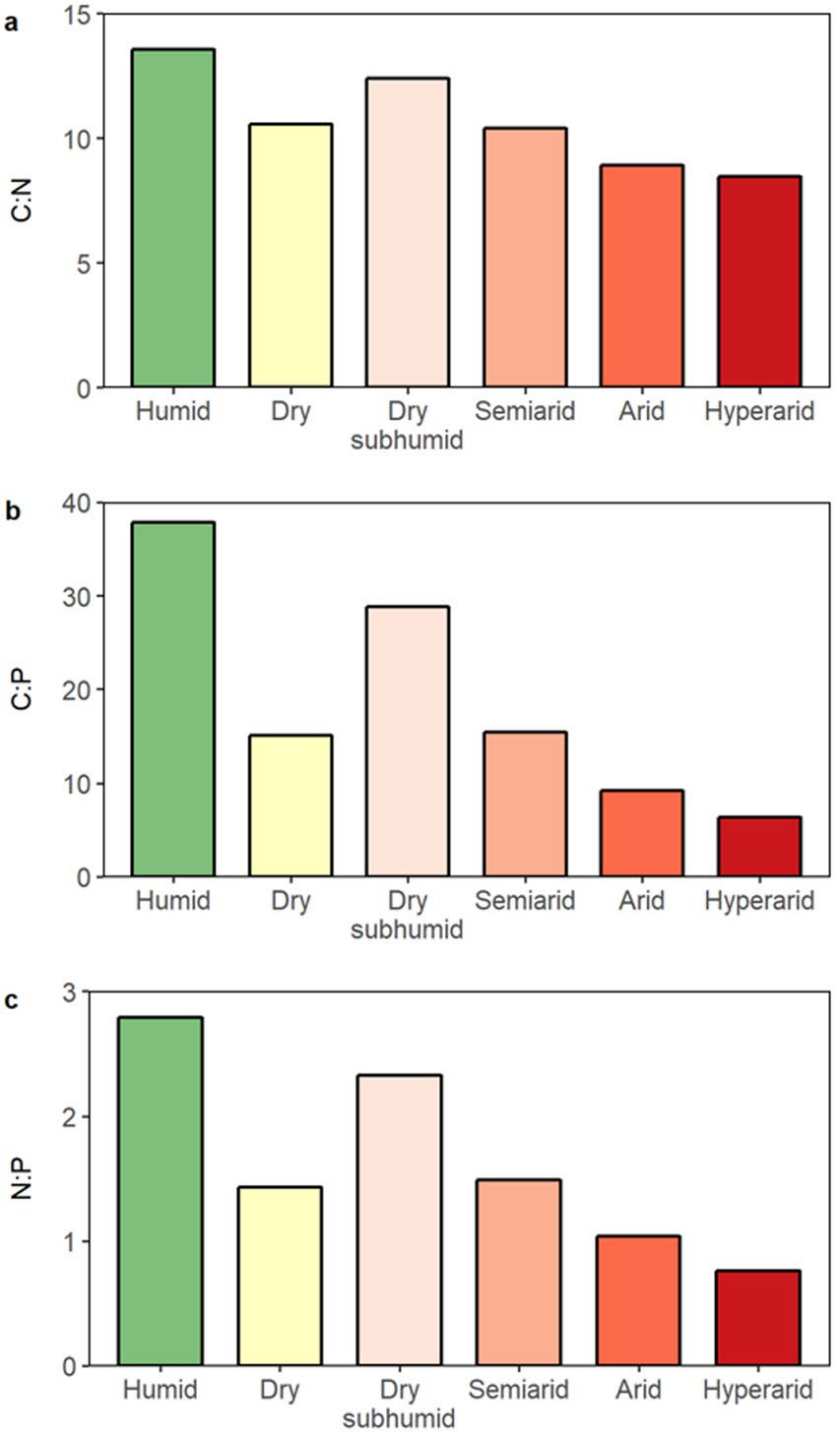
Soil C:N (**a**), C:P (**b**), and N:P (**c**) to 0.5-m depth in hyperarid, arid, semiarid, dry subhumid, dry, and humid lands.

Organic C:N, C:P, and N:P of dryland soils are smaller than those of soils from humid regions, and tend to decrease markedly with increasing aridity (Fig. 5). Specifically, the C:N:P ratio is 38:2.8:1 for mesic soils and 15:1.4:1 for dryland soils, and decreases from 29:2.3:1 for dry subhumid, to 15:1.5:1 for semiarid, to 9:1.0:1 for arid, and to 6:0.8:1 for hyperarid soils.

## Discussion

The distribution of major soils groups in drylands contrasts with that in humid regions, the latter being characterized by a higher diversity of more developed soil classes. Precipitation and temperature are well known to be among the most important factors that shape the formation of soils^24^. The relatively poor development of dryland soils can be explained by the inherently low and highly variable precipitation and high evapotranspiration. These climatic factors limit biological productivity and activity, chemical reactions, and weathering, while favoring soil surface erosion^25,26^.

Decreasing precipitation and increasing evapotranspiration reduce the loss of salts by leaching, leading to the accumulation of calcium carbonate and gypsum, high base saturation, and relatively high pH typical of dryland soils^27^. The coarser texture and low organic matter concentration of dryland soils compared to soils from humid regions explain their typically lower water storage capacity, higher bulk density, and lower cation exchange capacity^28^. As a whole, these properties results in low fertility and reinforce the effects of low water availability to limit biomass productivity and C sequestration. In fact, drylands cover 45% of the Earth’s land surface but only hold 16% of the C globally stored in vegetation (Supplementary Table S5 and Fig. S2).

Despite typically small content per unit area, the global amount of organic C stored in dryland soils is relevant. Our revised estimate of soil organic C stocks—470 ± 7 Pg to 1 m (646 ± 9 Pg to 2 m), or 32% of the global soil organic C pool—is greater than the 431 Pg, or 27% of the global reserves previously reported^1,18^. The dryland delimitation used in our study is based on more recent and detailed climatic data than those used for earlier reports^1,3,18^, and includes areas at northern high-latitude regions (i.e., north-western North America and north-eastern Asia) that were not accounted for previously as drylands^4^. These areas contain vast reserves of permafrost C formed from plant, animal, and microbial remains accumulating over thousands of years and protected from decomposition by frozen conditions^29^. Based on the WISE30sec soil database and the former delineation of boundaries used in previous reports, the mass of organic C stored in dryland soils would be estimated at 417 Pg. This indicates that the ascertained differences in organic C stocks between our estimates and previous ones can be attributed not only to differences in dryland extent but also to the soil geo-databases used for quantification. Our estimated quantity of organic C in dryland soils to 2 m is 38% smaller than the 1,035 ± 150 Pg found in permafrost soils in the uppermost 3 m^29^, 24% greater than the 520 ± 45 Pg in vegetation^30^, and 22% smaller than the 829 ± 10 Pg in the atmosphere^30^.

Recent modelling results indicate that human land use and cover change have caused the loss of 133 Pg of organic C from the upper 2 m of soil globally over the past 12,000 years, and there are hotspots of loss closely associated with major cropping regions and arid and semiarid degraded grazing lands^31^. Previous studies suggest that the historic soil organic C loss from drylands due to desertification is 19 to 29 Pg^32^. The sheer magnitude of this estimate, which would need updating, shows the potential to restore, preserve, and increase soil organic C in drylands. Building up soil organic matter by appropriate management systems is arguably the best strategy to improve essential intertwined soil properties, such as structure, water holding capacity, fertility, and resistance to erosion, and thus to enhance key dryland ecosystem functions and services, including primary production, food provision, biodiversity support, and climate change mitigation^12,26,33^. Biotic attributes, such as species richness and abundance, can be actively managed at the local scale for this purpose and to increase ecosystem resilience to global change drivers^34^. This can be achieved also by improved grazing regime and farming management, such as using organic amendments, mineral N fertilization, cover crops, crop rotations, shifting from conventional tillage to conservation tillage practices, and improving water use efficiency^12,35,36^. Compared to soils from humid regions, the effectiveness of such strategies on dryland soils may be limited by their typically coarser texture, with larger sand and smaller clay concentrations, as clay minerals provide soil organic matter protection from decomposition^13,37,38^.

Similar to our revised value of organic C stock, our estimate of soil inorganic C stock in drylands—1237 ± 15 Pg or about 80% of the global soil inorganic C—is significantly larger than the 916 Pg previously reported^1,18^, which can also be attributed to differences in dryland extent and soil geo-databases used for quantification. This quantity of C is 20% more than the C found in permafrost soils^29^, 138% more than that found in vegetation^30^, and 49% more than that in the atmosphere^30^. The soil inorganic C pool, mainly present as carbonates^39^, can be affected by land management practices, such as afforestation, irrigation, fertilization, and liming^40,41^. Practices leading to elevated carbon dioxide (CO_2_) in soils, such as those aimed at increasing soil organic matter content, have been found to result in significant formation and precipitation of secondary carbonates, thus contributing to soil C sequestration^40–42^. However, not only the size but also the sign of the effects of land management are still highly uncertain. Because of the magnitude of the amount of inorganic C stored in drylands soils and its potential to affect atmospheric CO_2_ concentration, these aspects warrant much more research attention.

In addition to C, we found significant amounts of N and especially P in dryland soils, for which there were no previous global estimates in the literature. We also found that aridity tends to decrease soil organic C and total N content to a different extent while increasing total and labile inorganic P content. This results in decreased C:N, C:P, and N:P, which agrees with previous studies reporting decoupled responses of C, N, and P cycles in drylands^21,43,44^. These findings may be related to increased release of P by rock physical weathering and reduced plant productivity and nutrient uptake with aridity^21^. Increased aridity and temperature may also decouple the spatial variability of soil nutrient stocks and cycling in global drylands^45^. Other than C, ecosystems need nutrients, especially N, to build up soil organic matter^46^. The typically low C:N and C:P ratios of dryland soils suggest that they are better suited than soils in humid regions for C sequestration.

The estimates of element stocks reported in this work are based on a limited dataset and are therefore uncertain. Knowledge of the estimation uncertainty is important for many reasons, if only to be able to assess whether observed changes in stocks over time are statistically significant. Similar to previous studies^22^, we quantified the uncertainties assuming that the estimation errors are spatially uncorrelated. As a result, a large part of the uncertainty cancels out when the estimates are spatially aggregated to a total stock for the entire dryland biome or the globe^47^. This explains why the standard deviations reported in Table 1 are unrealistically small. More realistic uncertainty estimates would have been obtained if spatial correlation would have been taken into account. Indeed, it has been shown that uncertainty estimates of spatial aggregates are extremely sensitive to the degree of spatial correlation of estimation errors^48^. Such geo-statistical analysis was beyond our current capacity and the scope of this study. Future work should aim to adopt and develop such approaches to obtain more realistic estimates of the uncertainties associated with global element stocks.

The datasets used here are the most recent and comprehensive soil inventories available at the global scale, yet important dryland zones, especially Australia, North America, South East Asia, and West Africa, are still underrepresented in current soil databases, and thus they may not accurately reflect current conditions^22^. Future efforts to enrich soil geo-databases with data from drylands will be critical to better understand the vulnerability of these areas to desertification and climate change, and to implement successful management practices to prevent and mitigate their impacts. For this purposes, it is also vital to keep updated the global aridity database, as proven in other studies related to land degradation^49^.

As a whole, our study shows that aridity results in soils with high vulnerability to erosion and degradation. Management practices aimed at building up soil organic matter may help mitigate climate change by sequestering C, and are essential to preserve and enhance other services of dryland ecosystems, such as biodiversity support, water regulation, and food production, with substantial global and local societal, economic, and environmental benefits. These practices may thus help to meet UN goals for sustainable development and the international initiative “4 per 1000”, aimed at increasing global soil organic C stocks by 0.4% per year to compensate for anthropogenic emissions of greenhouse gases^50^. The magnitude of historic soil C losses and the typically low ratios of C to other nutrients suggest potential to build up organic matter in dryland soils, but coarse soil textures together with the inherently low water availability pose limitations.

## Methods

### Spatial data

The geo-databases and maps used in this study included the CGIAR-CSI Global-Aridity and Global-PET Database^5^; the harmonized soil dataset of ISRIC-WISE (International Soil Reference and Information Centre-World Inventory of Soil Property Estimates) derived soil properties for the globe, or WISE30sec.^22^; and the Global Gridded Soil Phosphorus Distribution Maps at 0.5-degree Resolution^23^. Briefly, the CGIAR-CSI Global-Aridity Database models the annual average aridity index over the 1950–2000 period using WorldClim data^51^ on a global surface at a spatial resolution of 0.008333°. The WISE30sec dataset contains a number of soil physical and chemical properties for the globe, at seven layers to a depth of 2 m, and at a resolution of 0.008333°; WISE30sec was developed combining the soil map units of the Harmonized World Soil Database, version 1.2^52^, a map of Köppen-Geiger climate zones as co-variate, and ISRIC-WISE derived soil properties. The “Global Gridded Soil Phosphorus Distribution Maps at 0.5-degree Resolution” database provides estimates of labile inorganic, organic, occluded, secondary mineral, apatite, and total P contents in the top 50 cm of soil at a resolution of 0.5°. All spatial information was acquired from original sources. To the best of our knowledge, these are the most recent global geo-databases with the highest resolution that are currently available.

### GIS and data analysis

The CGIAR-CSI Global-Aridity raster was extracted by a mask of 1:10 m land polygons acquired from Natural Earth (Land polygons including major islands, version 3.0.1, and Islands that are 2 sq. km or less in size, version 3.0.0; http://www.naturalearthdata.com) and divided by aridity index (AI) into hyperarid (AI < 0.03), arid (0.03 ≤ AI < 0.20), semiarid (0.20 ≤ AI < 0.50), dry subhumid (0.50 ≤ AI < 0.65), dry (AI < 0.65), and humid (AI ≥ 0.65) zones. The resultant rasters were in turn used as masks to divide all the global maps used by AI zones. Eckert IV projection was used for area calculations. Soil C, N, and P stocks were calculated by aggregating contents (unit of mass of element per unit of area) of each map unit (grid cell) multiplied by its global area. Soil C and N contents were derived from the WISE30sec data of concentration, bulk density, gravel volume, and layer thickness, taking into account all the soil components of each map unit, using an approach similar to that described by Batjes^22^. We calculated the uncertainties associated with C and N stocks (STOCK_std_, kg m^−2^) from the layer thickness (HOT, m), concentration (CN, g kg^−1^), bulk density (BULK, g cm^−3^), gravel volume (CFRAG, %), and their corresponding standard deviations (std) reported in WISE30sec using the Taylor series method^53^.

$$STOC{K}_{std}=HOT\times \sqrt{\begin{array}{c}C{{N}_{std}}^{2}\times BUL{K}^{2}\times {(1-\frac{CFRAG}{100})}^{2}+\\ C{N}^{2}\times BUL{{K}_{std}}^{2}\times {(1-\frac{CFRAG}{100})}^{2}+\\ C{N}^{2}\times BUL{K}^{2}\times {(\frac{CFRA{G}_{std}}{100})}^{2}\end{array}}$$

ArcGIS 10.5 for Desktop (Esri Inc., Redlands, CA) was used for processing rasters and creating maps, whereas major attribute data processing, statistical summaries, and plots were performed using R 3.5.1^54^ and the R packages RODBC^55^, dplyr^56^, Hmisc^57^, xlsx^58^, and ggplot2^59^.

## Electronic supplementary material

Supplementary Information

## Data Availability

The data and R scripts related to this study are available from the corresponding author upon request.
