# Supplementary material for: Soil resources and element stocks in drylands to face global issues
Source: Sci Rep. 2018 Sep 13;8:13788. doi: 10.1038/s41598-018-32229-0 (PMC6137228; doi:10.1038/s41598-018-32229-0)
Supplement: Supplementary file 1 — Supplementary Information [file 41598_2018_32229_MOESM1_ESM.pdf]

Supplementary Information

Soil resources and element stocks in drylands to face global issues

César Plaza*, Claudio Zaccone, Kasia Sawicka, Ana M. Méndez, Ana Tarquis, Gabriel Gascó, Gerard B. M. Heuvelink, Edward A. G. Schuur, Fernando T. Maestre

This file includes:

- Tables S1 to S5
- Figures S1 and S2
- Additional references

Table S1. Extent, area used for cultivating crops and pasture, and human population of hyperarid, arid, semiarid, dry subhumid, dry and humid areas. Data derived from CGIAR-CSI Global-Aridity and Global-PET Database[5]; IIASA-IFPRI Global Cropland Map[60]; Global Agricultural Lands: Pastures, 2000[61]; Gridded Population of the World, Version 4 (GPWv4): UN-Adjusted Population Count, v4, 2015[62] using the approach described in the Methods section.

Land	Total area		Cropland area		Pasture area		Population		
	Mkm^2	% of global	Mkm^2	% of global	Mkm^2	% of global	Billion	People per km^{-2}	% of global
Hyperarid	8.6	5.8	0.04	0.2	0.36	1.3	0.09	10.6	1.3
Arid	20.8	14.0	0.85	5.6	6.65	24.5	0.48	23.0	6.6
Semiarid	24.1	16.1	4.01	26.4	10.13	37.3	1.31	54.5	18.0
Dry subhumid	13.2	8.9	2.73	18.0	2.88	10.6	0.96	73.0	13.2
Dry	66.7	44.8	7.63	50.1	20.02	73.6	2.84	42.6	39.0
Humid	82.2	55.2	7.58	49.9	7.17	26.4	4.44	54.0	61.0
Global	149[63]	100	15.21	100	27.19	100	7.29	48.9	100

Table S2. Proportion of area covered by major soil groups in hyperarid, arid, semiarid, dry subhumid, dry and humid areas. Data derived from the WISE30sec[22] dataset using the approach described in the Methods section.

Hyperarid		Arid		Semiarid		Dry subhumid		Dry		Humid		Global	
Soil group	%	Soil group	%	Soil group	%	Soil group	%	Soil group	%	Soil group	%	Soil group	%
Regosols	25.78	Regosols	18.76	Leptosols	12.92	Cambisols	13.33	Regosols	14.66	Cambisols	10.72	Leptosols	11.45
Sand dunes	19.38	Calcisols	16.92	Kastanozems	11.33	Leptosols	11.22	Leptosols	13.28	Acrisols	10.59	Regosols	10.61
Leptosols	17.32	Arenosols	13.50	Regosols	10.79	Regosols	7.98	Arenosols	9.28	Leptosols	9.67	Cambisols	8.57
Arenosols	12.26	Leptosols	13.32	Luvisols	9.56	Luvisols	7.30	Calcisols	8.38	Ferralsols	8.43	Luvisols	6.25
Calcisols	7.40	Luvisols	6.20	Cambisols	8.01	Gleysols	7.29	Luvisols	6.93	Podzols	8.20	Arenosols	5.84
Rock outcrops	5.64	Solonchaks	5.37	Arenosols	7.65	Chernozems	7.02	Cambisols	6.38	Gleysols	8.17	Acrisols	5.75
Gypsisols	3.58	Gypsisols	4.67	Vertisols	6.42	Phaeozems	4.75	Kastanozems	4.99	Regosols	6.64	Gleysols	5.44
Fluvisols	3.16	Sand dunes	4.67	Calcisols	5.76	Fluvisols	4.38	Sand dunes	4.16	Luvisols	5.58	Ferralsols	4.82
Solonchaks	3.09	Solonetz	3.50	Solonetz	4.92	Lixisols	4.20	Vertisols	3.54	Histosols	4.38	Podzols	4.49
Luvisols	0.77	Fluvisols	2.63	Fluvisols	3.12	Ferralsols	3.76	Solonetz	3.35	Glaciers	3.42	Calcisols	4.18
Salt flats	0.63	Cambisols	2.50	Phaeozems	2.78	Arenosols	3.68	Fluvisols	3.22	Podzoluvisols	3.18	Fluvisols	3.13
Cambisols	0.50	Vertisols	1.81	Gleysols	2.53	Vertisols	3.24	Gleysols	2.65	Fluvisols	3.04	Kastanozems	2.55
Phaeozems	0.10	Rock outcrops	1.30	Lixisols	2.48	Kastanozems	3.14	Solonchaks	2.62	Arenosols	2.48	Histosols	2.46
Vertisols	0.10	Gleysols	0.90	Chernozems	2.29	Acrisols	2.69	Chernozems	2.23	Phaeozems	2.03	Vertisols	2.12
Water bodies	0.10	Kastanozems	0.88	Planosols	2.06	Podzols	2.49	Phaeozems	2.07	Nitisols	1.85	Sand dunes	2.08
Gleysols	0.08	Water bodies	0.81	Solonchaks	1.29	Solonetz	2.38	Gypsisols	2.02	Lixisols	1.47	Phaeozems	2.05
Anthrosols	0.04	Planosols	0.54	Ferralsols	1.07	Histosols	2.00	Lixisols	1.78	Plinthosols	1.34	Solonetz	1.87
Solonetz	0.03	Salt flats	0.41	Acrisols	0.77	Water bodies	1.70	Rock outcrops	1.38	Andosols	1.25	Glaciers	1.78
Planosols	0.03	Phaeozems	0.35	Water bodies	0.64	Planosols	1.41	Planosols	1.19	Water bodies	1.15	Podzoluvisols	1.74
Andosols	0.02	Anthrosols	0.33	Podzols	0.59	Nitisols	1.35	Ferralsols	1.14	Chernozems	0.95	Lixisols	1.62
Kastanozems	0.00	Andosols	0.23	Sand dunes	0.56	Podzoluvisols	1.28	Water bodies	0.83	Rock outcrops	0.88	Chernozems	1.58
Acrisols	0.00	Lixisols	0.16	Nitisols	0.46	Greyzems	0.91	Acrisols	0.82	Planosols	0.82	Solonchaks	1.37
Alisols	0.00	Glaciers	0.09	Rock outcrops	0.46	Plinthosols	0.53	Podzols	0.71	Greyzems	0.78	Nitisols	1.15
Chernozems	0.00	Chernozems	0.05	Histosols	0.32	Rock outcrops	0.41	Histosols	0.52	Alisols	0.77	Rock outcrops	1.13
Ferralsols	0.00	Acrisols	0.03	Gypsisols	0.29	Andosols	0.39	Nitisols	0.44	Vertisols	0.74	Planosols	1.00
Fish ponds	0.00	Alisols	0.02	Anthrosols	0.22	Solonchaks	0.39	Podzoluvisols	0.27	Anthrosols	0.62	Gypsisols	1.00
Glaciers	0.00	Nitisols	0.02	Andosols	0.21	Calcisols	0.37	Greyzems	0.23	Solonetz	0.43	Water bodies	1.00
Greyzems	0.00	Podzols	0.01	Glaciers	0.15	Glaciers	0.15	Andosols	0.23	Kastanozems	0.16	Andosols	0.74

Histosols	0.00	Ferralsols	0.01	Greyzems	0.13	Alisols	0.12	Salt flats	0.22	Solonchaks	0.15	Plinthosols	0.74
Humanly disturbed	0.00	Histosols	0.01	Plinthosols	0.08	Anthrosols	0.08	Anthrosols	0.21	Calcisols	0.05	Greyzems	0.51
Island	0.00	Greyzems	0.00	Alisols	0.06	Urban, mining, etc.	0.01	Plinthosols	0.13	Urban, mining, etc.	0.04	Anthrosols	0.42
Lixisols	0.00	Fish ponds	0.00	Podzoluvisols	0.04	Sand dunes	0.01	Glaciers	0.11	Sand dunes	0.03	Alisols	0.42
Nitisols	0.00	Island	0.00	Salt flats	0.02	Fish ponds	0.01	Alisols	0.05	Island	0.00	Salt flats	0.11
Plinthosols	0.00	Humanly disturbed	0.00	Urban, mining, etc.	0.00	Gypsisols	0.00	Urban, mining, etc.	0.00	Gypsisols	0.00	Urban, mining, etc.	0.02
Podzols	0.00	Podzoluvisols	0.00	Fish ponds	0.00	Humanly disturbed	0.00	Fish ponds	0.00	Fish ponds	0.00	Fish ponds	0.00
Podzoluvisols	0.00	Plinthosols	0.00	Island	0.00	Island	0.00	Island	0.00	Humanly disturbed	0.00	Island	0.00
Urban, mining, etc.	0.00	Urban, mining, etc.	0.00	Humanly disturbed	0.00	Salt flats	0.00	Humanly disturbed	0.00	Salt flats	0.00	Humanly disturbed	0.00

Table S3. Physical and chemical properties of topsoils (0-20 cm) in hyperarid, arid, semiarid, dry subhumid, dry and humid areas. Data derived from the WISE30sec dataset[22] using the approach described in the Methods section. Min, minimum; Q1, first quartile; Q3, third quartile; Max, maximum.

Property	Land	Min	Q1	Median	Mean	Q3	Max
Available water capacity	Hyperarid	6.0	12.0	13.0	14.0	18.0	25.0
(cm m^{-1})	Arid	6.0	13.0	15.0	14.6	18.0	40.0
	Semiarid	6.0	13.0	16.0	15.4	18.0	45.0
	Dry subhumid	6.0	14.0	16.0	17.0	19.0	45.0
	Dry	6.0	13.0	15.0	15.3	18.0	45.0
	Humid	6.0	14.0	17.0	18.3	22.0	45.0
	Global	6.0	13.0	16.0	16.8	19.0	45.0
Gravel content	Hyperarid	1.0	7.0	12.0	12.3	18.0	46.0
(% v/v)	Arid	1.0	6.0	12.0	11.1	17.0	46.0
	Semiarid	0.0	5.0	11.0	11.1	17.0	46.0
	Dry subhumid	0.0	4.0	11.0	12.2	18.0	46.0
	Dry	0.0	6.0	11.0	11.5	17.0	46.0
	Humid	0.0	4.0	11.0	12.6	16.0	49.0
	Global	0.0	5.0	11.0	12.1	17.0	49.0
Sand fraction	Hyperarid	18.0	55.0	57.0	62.7	66.0	90.0
(%)	Arid	11.0	49.0	55.0	58.9	66.0	92.0
	Semiarid	9.0	38.0	49.0	50.6	64.0	94.0
	Dry subhumid	9.0	33.0	40.0	44.7	53.0	94.0
	Dry	9.0	41.0	51.0	53.2	65.0	94.0
	Humid	8.0	36.0	43.0	46.3	54.0	94.0
	Global	8.0	37.0	49.0	49.7	59.0	94.0
Silt fraction	Hyperarid	4.0	20.0	26.0	23.2	27.0	46.0
(%)	Arid	4.0	20.0	26.0	24.2	31.0	54.0
	Semiarid	4.0	21.0	29.0	27.8	36.0	67.0
	Dry subhumid	4.0	24.0	33.0	33.5	44.0	67.0
	Dry	4.0	21.0	28.0	27.4	33.0	67.0
	Humid	4.0	21.0	31.0	32.1	42.0	67.0
	Global	4.0	21.0	29.0	29.8	38.0	67.0
Clay fraction	Hyperarid	4.0	12.0	16.0	14.1	16.0	55.0
(%)	Arid	3.0	12.0	16.0	16.9	19.0	65.0
	Semiarid	2.0	15.0	20.0	21.5	24.0	65.0
	Dry subhumid	2.0	15.0	20.0	21.8	26.0	65.0
	Dry	2.0	14.0	18.0	19.4	23.0	65.0
	Humid	2.0	14.0	20.0	21.6	27.0	65.0
	Global	2.0	14.0	19.0	20.5	24.0	65.0
Bulk density	Hyperarid	0.81	1.37	1.43	1.42	1.45	1.64
(g cm^{-3})	Arid	0.14	1.39	1.45	1.44	1.48	1.76
	Semiarid	0.11	1.37	1.44	1.42	1.48	1.76
	Dry subhumid	0.11	1.25	1.37	1.30	1.43	1.76

	Dry	0.11	1.37	1.43	1.40	1.47	1.76
	Humid	0.11	1.13	1.30	1.23	1.38	1.76
	Global	0.11	1.26	1.37	1.31	1.45	1.76
pH (H_2O)	Hyperarid	4.8	7.4	7.8	7.6	8.0	8.6
	Arid	4.3	7.0	7.7	7.5	8.2	8.6
	Semiarid	4.3	6.4	7.0	6.9	7.6	9.0
	Dry subhumid	4.0	5.4	6.3	6.3	6.8	9.0
	Dry	4.0	6.4	7.3	7.0	7.9	9.0
	Humid	4.0	5.0	5.3	5.6	6.2	9.0
	Global	4.0	5.2	6.3	6.3	7.4	9.0
Cation exchange capacity of clay fraction (cmol kg^{-1})	Hyperarid	20.0	47.0	63.0	59.4	74.0	127.0
	Arid	8.0	38.0	64.0	56.9	68.0	128.0
	Semiarid	7.0	42.0	59.0	54.9	67.0	128.0
	Dry subhumid	2.0	35.0	51.0	48.4	63.0	128.0
	Dry	2.0	39.0	59.0	54.6	67.0	128.0
	Humid	2.0	20.0	44.0	41.4	57.0	128.0
	Global	2.0	34.0	49.0	47.9	66.0	128.0
Cation exchange capacity of soil (cmol kg^{-1})	Hyperarid	2.0	6.0	13.0	10.4	13.0	40.0
	Arid	2.0	6.0	13.0	12.3	14.0	86.0
	Semiarid	2.0	12.0	15.0	16.8	20.0	105.0
	Dry subhumid	2.0	12.0	18.0	20.9	26.0	105.0
	Dry	2.0	10.0	14.0	15.6	18.0	105.0
	Humid	2.0	9.0	16.0	20.9	24.0	105.0
	Global	2.0	10.0	15.0	18.3	20.0	105.0
Effective cation exchange capacity (cmol kg^{-1})	Hyperarid	2.0	20.0	27.0	29.8	37.0	143.0
	Arid	2.0	15.0	30.0	29.3	38.0	143.0
	Semiarid	1.0	13.0	23.0	23.0	31.0	143.0
	Dry subhumid	1.0	10.0	18.0	18.6	26.0	143.0
	Dry	1.0	13.0	25.0	24.7	33.0	143.0
	Humid	1.0	6.0	11.0	14.4	21.0	143.0
	Global	1.0	7.0	15.0	19.5	27.0	143.0
Al saturation (%)	Hyperarid	0.0	0.0	0.0	0.0	0.0	85.0
	Arid	0.0	0.0	0.0	1.1	0.0	51.0
	Semiarid	0.0	0.0	0.0	2.1	0.0	85.0
	Dry subhumid	0.0	0.0	0.0	6.2	9.0	85.0
	Dry	0.0	0.0	0.0	2.4	0.0	85.0
	Humid	0.0	0.0	11.0	13.8	21.0	85.0
	Global	0.0	0.0	0.0	8.2	14.0	85.0
Base saturation (%)	Hyperarid	29.0	89.0	98.0	90.3	100.0	100.0
	Arid	10.0	82.0	98.0	88.8	100.0	100.0
	Semiarid	10.0	77.0	91.0	84.4	99.0	100.0
	Dry subhumid	10.0	61.0	79.0	72.5	91.0	100.0
	Dry	10.0	75.0	91.0	83.9	99.0	100.0

	Humid	8.0	34.0	56.0	55.9	77.0	100.0
	Global	8.0	45.0	77.0	69.7	93.0	100.0
Exchangeable Na	Hyperarid	1.0	3.0	3.0	4.7	3.0	67.0
(%)	Arid	1.0	2.0	3.0	6.3	4.0	73.0
	Semiarid	1.0	1.0	2.0	3.9	3.0	73.0
	Dry subhumid	0.0	1.0	2.0	2.4	2.0	73.0
	Dry	0.0	2.0	2.0	4.4	3.0	73.0
	Humid	0.0	1.0	2.0	2.0	2.0	73.0
	Global	0.0	1.0	2.0	3.2	3.0	73.0
Calcium carbonate	Hyperarid	0.0	44.0	63.0	71.1	84.0	273.0
concentration	Arid	0.0	18.0	63.0	68.9	99.0	313.0
(g kg^{-1})	Semiarid	0.0	0.0	17.0	39.3	63.0	313.0
	Dry subhumid	0.0	0.0	0.0	22.2	0.0	286.0
	Dry	0.0	0.0	28.0	48.2	80.0	313.0
	Humid	0.0	0.0	0.0	10.0	0.0	313.0
	Global	0.0	0.0	0.0	28.8	45.0	313.0
Gypsum concentration	Hyperarid	0.0	2.0	3.0	38.6	40.0	576.0
(g kg^{-1})	Arid	0.0	1.0	3.0	33.8	33.0	576.0
	Semiarid	0.0	1.0	2.0	8.8	8.0	576.0
	Dry subhumid	0.0	0.0	3.0	6.6	8.0	454.0
	Dry	0.0	1.0	3.0	19.1	16.0	576.0
	Humid	0.0	0.0	0.0	4.7	3.0	576.0
	Global	0.0	0.0	1.0	11.8	8.0	576.0
Electrical conductivity	Hyperarid	0.0	1.0	1.0	1.5	1.0	32.0
(dS m^{-1})	Arid	0.0	1.0	1.0	1.7	1.0	32.0
	Semiarid	0.0	0.0	1.0	1.0	1.0	32.0
	Dry subhumid	0.0	0.0	1.0	0.9	1.0	32.0
	Dry	0.0	1.0	1.0	1.2	1.0	32.0
	Humid	0.0	0.0	1.0	0.6	1.0	32.0
	Global	0.0	0.0	1.0	0.9	1.0	32.0
Organic C concentration	Hyperarid	2.4	5.8	6.2	5.8	7.0	65.7
(g kg^{-1})	Arid	1.7	5.6	6.4	7.5	7.0	425.2
	Semiarid	1.7	6.2	9.3	15.8	17.0	496.8
	Dry subhumid	1.7	11.4	17.1	36.8	31.6	496.8
	Dry	1.7	5.9	7.1	16.6	16.0	496.8
	Humid	1.7	13.6	19.5	48.4	40.1	496.8
	Global	1.7	7.0	14.2	32.7	25.7	496.8
Total N concentration	Hyperarid	0.2	0.5	0.7	0.6	0.8	4.6
(g kg^{-1})	Arid	0.2	0.6	0.7	0.8	0.8	14.7
	Semiarid	0.2	0.7	1.0	1.3	1.5	23.5
	Dry subhumid	0.2	1.1	1.5	2.3	2.3	23.5
	Dry	0.2	0.6	0.8	1.3	1.5	23.5
	Humid	0.2	1.1	1.5	2.7	2.7	23.5

	Global	0.2	0.8	1.3	2.0	1.9	23.5
C:N ratio	Hyperarid	8.0	9.0	9.0	10.1	11.0	16.0
	Arid	8.0	9.0	9.0	10.1	11.0	25.0
	Semiarid	7.0	9.0	11.0	11.0	12.0	29.0
	Dry subhumid	7.0	11.0	12.0	12.9	13.0	29.0
	Dry	7.0	9.0	11.0	11.0	12.0	29.0
	Humid	7.0	12.0	12.0	13.9	15.0	29.0
	Global	7.0	10.0	12.0	12.5	13.0	29.0

Table S4. Stock and density of different forms of soil P in hyperarid, arid, semiarid, dry subhumid, dry and humid areas. Data derived from the Global Gridded Soil Phosphorus Distribution Maps at 0.5-degree Resolution[23] using the approach described in the Methods section. Min, minimum; Q1, first quartile; Q3, third quartile; Max, maximum.

P form	Land	Stock (Pg)	Density (kg m^{-2})					
			Min	Q1	Median	Mean	Q3	Max
Total P	Hyperarid	2.41	0.112	0.272	0.322	0.391	0.485	1.324
	Arid	6.76	0.112	0.297	0.297	0.408	0.448	1.577
	Semiarid	8.79	0.045	0.297	0.322	0.417	0.504	1.577
	Dry subhumid	4.20	0.045	0.272	0.322	0.391	0.504	1.577
	Dry	22.16	0.045	0.297	0.322	0.406	0.485	1.577
	Humid	17.62	0.045	0.207	0.297	0.340	0.426	1.577
	Global	39.78	0.045	0.254	0.322	0.374	0.448	1.577
Labile inorganic P	Hyperarid	0.25	0.008	0.023	0.035	0.041	0.043	0.143
	Arid	0.65	0.007	0.025	0.025	0.039	0.040	0.170
	Semiarid	0.74	0.003	0.019	0.026	0.035	0.040	0.195
	Dry subhumid	0.35	0.003	0.016	0.026	0.033	0.039	0.195
	Dry	1.99	0.003	0.022	0.026	0.037	0.040	0.195
	Humid	1.51	0.003	0.014	0.022	0.029	0.037	0.195
	Global	3.50	0.003	0.017	0.025	0.033	0.038	0.195
Organic P	Hyperarid	0.32	0.005	0.025	0.049	0.051	0.049	0.382
	Arid	0.75	0.005	0.014	0.024	0.045	0.049	0.538
	Semiarid	1.62	0.005	0.036	0.056	0.077	0.097	0.641
	Dry subhumid	0.96	0.005	0.048	0.074	0.089	0.125	0.641
	Dry	3.65	0.005	0.024	0.049	0.067	0.083	0.641
	Humid	4.75	0.005	0.049	0.074	0.092	0.125	0.641
	Global	8.40	0.005	0.032	0.056	0.079	0.106	0.641
Occluded P	Hyperarid	0.50	0.019	0.047	0.069	0.081	0.087	0.286
	Arid	1.41	0.019	0.051	0.069	0.085	0.087	0.410
	Semiarid	2.68	0.019	0.069	0.112	0.127	0.150	0.687
	Dry subhumid	1.41	0.019	0.073	0.112	0.131	0.169	0.687
	Dry	6.00	0.019	0.051	0.081	0.110	0.131	0.687
	Humid	6.02	0.019	0.069	0.104	0.116	0.131	0.687
	Global	12.03	0.019	0.069	0.103	0.113	0.131	0.687
Secondary mineral P	Hyperarid	0.13	0.006	0.016	0.017	0.021	0.026	0.070
	Arid	0.38	0.006	0.017	0.017	0.023	0.026	0.099
	Semiarid	0.58	0.005	0.017	0.021	0.028	0.034	0.166
	Dry subhumid	0.34	0.005	0.016	0.028	0.031	0.041	0.166
	Dry	1.43	0.005	0.017	0.017	0.026	0.031	0.166
	Humid	1.67	0.005	0.017	0.028	0.032	0.042	0.166
	Global	3.10	0.005	0.017	0.023	0.029	0.034	0.166
Apatite P	Hyperarid	1.21	0.021	0.152	0.152	0.197	0.229	0.624
	Arid	3.57	0.001	0.152	0.189	0.215	0.231	0.926
	Semiarid	3.16	0.0004	0.082	0.100	0.150	0.189	0.926

Dry subhumid	1.14	0.0004	0.051	0.082	0.106	0.147	0.743
Dry	9.09	0.0004	0.082	0.152	0.167	0.189	0.926
Humid	3.67	0.0004	0.007	0.051	0.071	0.095	0.743
Global	12.75	0.0004	0.042	0.086	0.120	0.163	0.926

Table S5. Stock and content of aboveground C in living vegetation (biomass C) in hyperarid, arid, semiarid, dry subhumid, dry and humid areas. Data derived from Global Aboveground Biomass Carbon, Version 1.0[64] using the approach described in the Method section. Min, minimum; Q1, first quartile; Q3, third quartile; Max, maximum.

Land	Biomass C stock (Pg)	Biomass C content (kg m^{-2})					
		Min	Q1	Median	Mean	Q3	Max
Hyperarid	1	0.0	0.0	0.0	0.2	0.3	2.6
Arid	8	0.0	0.3	0.4	0.4	0.5	15.0
Semiarid	23	0.0	0.4	0.6	1.0	1.0	15.0
Dry subhumid	26	0.0	0.5	1.1	2.0	2.5	14.6
Dry	59	0.0	0.3	0.5	0.9	0.8	15.0
Humid	312	0.0	0.9	3.1	4.9	8.6	15.0
Global	371	0.0	0.4	0.8	2.9	3.5	15.0

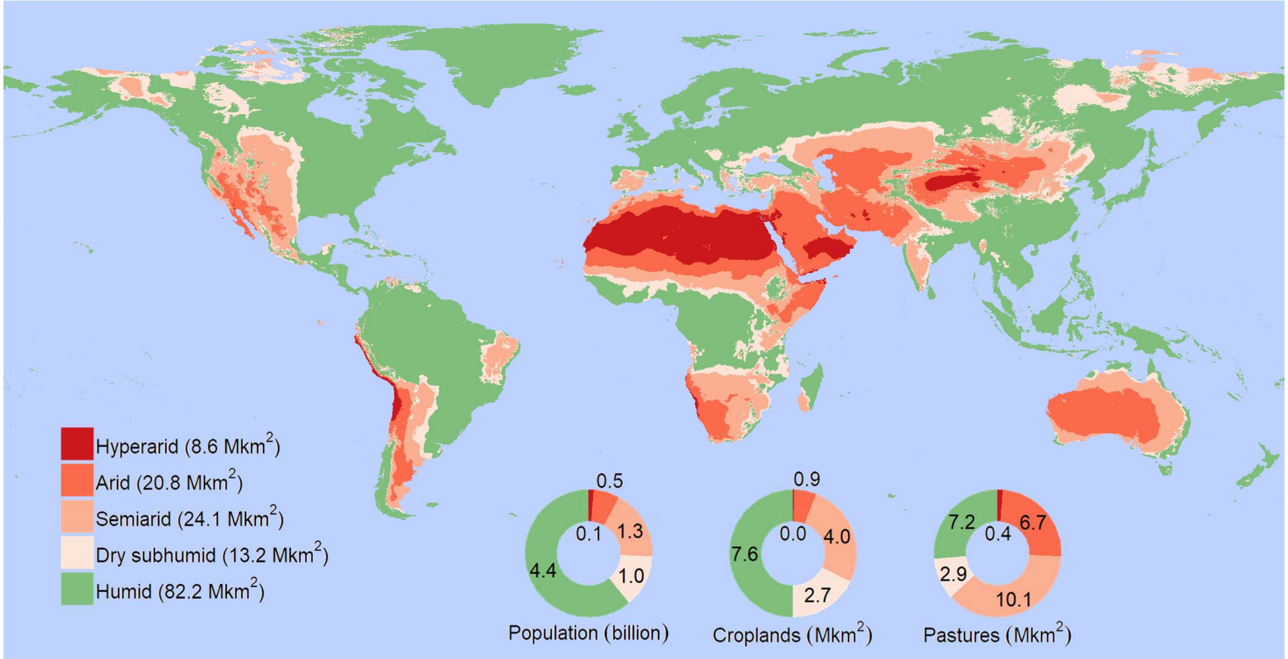

Figure S1. Global map of drylands and distribution of population and areas devoted to crop and pasture. Based on the aridity index (AI), or ratio of total annual precipitation to potential evapotranspiration, drylands are divided into hyperarid (AI less than 0.03 mm mm^{-1}), arid (AI within the rage from 0.03 to 0.2 mm mm^{-1}), semiarid (AI from 0.2 to 0.5 mm mm^{-1}), and dry subhumid regions (AI from 0.5 to 0.65 mm mm^{-1}). Data derived from CGIAR-CSI Global-Aridity and Global-PET Database[1]; IIASA-IFPRI Global Cropland Map[5]; Global Agricultural Lands: Pastures, 2000[61]; Gridded Population of the World, Version 4 (GPWv4): UN-Adjusted Population Count, v4, 2015[62] using the approach described in the Methods section.

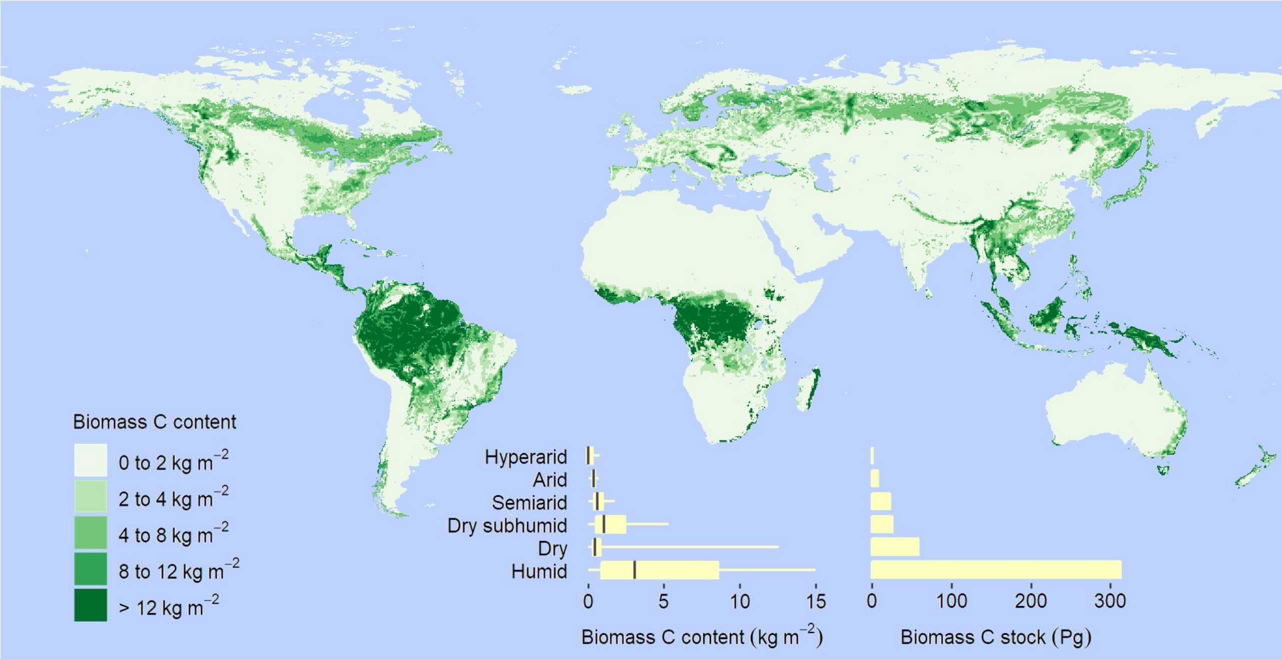

Figure S2. Global distribution of content and stock of aboveground C in living vegetation (biomass C). Based on the aridity index (AI), or ratio of total annual precipitation to potential evapotranspiration, drylands are divided into hyperarid (AI less than 0.03 mm mm^{-1}), arid (AI within the rage from 0.03 to 0.2 mm mm^{-1}), semiarid (AI from 0.2 to 0.5 mm mm^{-1}), and dry subhumid regions (AI from 0.5 to 0.65 mm mm^{-1}). Box, first and third quartile; central horizontal line, median; whisker, 1.5 times the interquartile range, or maximum or minimum if less. Data derived from Global Aboveground Biomass Carbon, Version 1.0[64] using the approach described in the Methods section.

Additional references

60. Fritz, S. *et al.* Mapping global cropland and field size. *Glob. Change Biol.* **21,** 1980-1992 (2015).

61. Ramankutty, N., Evan, A. T., Monfreda, C. & Foley, J. A. Global Agricultural Lands: Pastures, 2000. Socioeconomic Data and Applications Center (SEDAC). http://sedac.ciesin.columbia.edu/es/aglands.html (2010).

62. CIESIN - Columbia University. Gridded Population of the World, Version 4 (GPWv4): Population Count Adjusted to Match 2015 Revision of UN WPP Country Totals. NASA Socioeconomic Data and Applications Center (SEDAC). http://dx.doi.org/10.7927/H4SF2T42 (2016).

63. CIA. The World Factbook 2017. https://www.cia.gov/library/publications/the-world-factbook/index.html (2017).

64. Liu, Y. Y. *et al.* Recent reversal in loss of global terrestrial biomass. *Nat. Clim. Change* **5,** 470-474 (2015).
